# Iron Status and Cancer Risk in UK Biobank: A Two-Sample Mendelian Randomization Study

**DOI:** 10.3390/nu12020526

**Published:** 2020-02-19

**Authors:** Shuai Yuan, Paul Carter, Mathew Vithayathil, Siddhartha Kar, Edward Giovannucci, Amy M. Mason, Stephen Burgess, Susanna C. Larsson

**Affiliations:** 1 Unit of Cardiovascular and Nutritional Epidemiology, Institute of Environmental Medicine, Karolinska Institutet, SE-171 77 Stockholm, Sweden; shuai.yuan@ki.se; 2 Department of Surgical Sciences, Uppsala University, SE-751 85 Uppsala, Sweden; 3 Department of Public Health and Primary Care, University of Cambridge, Cambridge CB1 8RN, UK; paul_richard_carter@outlook.com (P.C.); siddhartha.kar@bristol.ac.uk (S.K.); am2609@medschl.cam.ac.uk (A.M.M.); sb452@medschl.cam.ac.uk (S.B.); 4 MRC Cancer Unit, University of Cambridge, Cambridge CB2 0XZ, UK; mat2k89@gmail.com; 5 MRC Integrative Epidemiology Unit, Bristol Medical School, University of Bristol, Bristol BS8 1QU, UK; 6 Departments of Epidemiology and Nutrition, Harvard T. H. Chan School of Public Health, Boston, MA 02115, USA; egiovann@hsph.harvard.edu; 7 Channing Division of Network Medicine, Department of Medicine, Brigham and Women’s Hospital, and Harvard Medical School, Boston, MA 02115, USA; 8 MRC Biostatistics Unit, University of Cambridge, Cambridge CB2 0SR, UK

**Keywords:** iron, transferrin saturation, ferritin, serum transferrin, cancer, Mendelian randomization

## Abstract

We conducted a two-sample Mendelian randomization study to explore the associations of iron status with overall cancer and 22 site-specific cancers. Single-nucleotide polymorphisms for iron status were obtained from a genome-wide association study of 48,972 European-descent individuals. Summary-level data for breast and other cancers were obtained from the Breast Cancer Association Consortium and UK Biobank. Genetically predicted iron status was positively associated with liver cancer and inversely associated with brain cancer but not associated with overall cancer or the other 20 studied cancer sites at *p* < 0.05. The odds ratios of liver cancer were 2.45 (95% CI, 0.81, 7.45; *p* = 0.11), 2.11 (1.16, 3.83; *p* = 0.02), 10.89 (2.44, 48.59; *p* = 0.002) and 0.30 (0.17, 0.53; *p* = 2 × 10^−5^) for one standard deviation increment of serum iron, transferrin saturation, ferritin and transferrin levels, respectively. For brain cancer, the corresponding odds ratios were 0.69 (0.48, 1.00; *p* = 0.05), 0.75 (0.59, 0.97; *p* = 0.03), 0.41 (0.20, 0.88; *p* = 0.02) and 1.49 (1.04, 2.14; *p* = 0.03). Genetically high iron status was positively associated with liver cancer and inversely associated with brain cancer.

## 1. Introduction 

Hereditary hemochromatosis, an autosomal recessive disorder characterized by a progressive iron overload, greatly increases the risk of developing hepatocellular carcinoma and non-neoplastic liver diseases [1,2]. However, the carcinogenic effects of physiologically high levels of iron, an essential nutrient influencing cell proliferation and growth [3], on liver cancer and non-hepatic malignancies are unknown. 

A randomized trial of 1277 individuals with peripheral arterial disease (636 in iron reduction group through venesection and 641 in control group) found that in the iron reduction group, who received regular phlebotomy, the risk of overall cancer, cancer-specific mortality and all-cause mortality was lower than in the control group after 4.5 years of follow-up [4]. However, findings of observational studies are inconsistent or scarce concerning the effects of iron status on individual cancers, such as colorectal, breast and oesophagal cancer [5,6,7,8,9,10,11]. Consequently, whether iron status (which is routinely measured in clinical practice as serum iron, transferrin saturation, ferritin and transferrin) plays a role in the development of site-specific cancer remains inconclusive. 

Mendelian randomization (MR) is an approach that can strengthen the inference about the causal nature of exposure–outcome associations by exploiting genetic variants as instrumental variables of the exposures [12]. This method has the strength of minimizing confounding as genetic variants are randomly assorted at conception, thereby being irrelevant to self-selected lifestyle and environmental factors. Additionally, it overcomes reverse causality since allelic randomization antedates the disease’s development. We conducted a two-sample MR study to systematically assess the possible causal associations of four iron status biomarkers with risk of overall and 22 site-specific cancers. 

## 2. Methods

### 2.1. Study Design Overview

We used a two-sample MR approach by using summary statistics data from a genome-wide association study (GWAS) of iron status, the Breast Cancer Association Consortium and the UK Biobank, which obtained appropriate patient consent and ethical approval. Overall cancer was treated as the primary outcome and the site-specific cancers as secondary outcomes. Information on the studies and consortia used in the present study is summarized in Supplementary Table S1. The present study was approved by the Swedish Ethical Review Authority. 

### 2.2. Instrumental Variable Selection

In a GWAS consisting of 48,972 individuals of European ancestry, the Genetics of Iron Status Consortium identified five single nucleotide polymorphisms (SNP) associated with serum iron and transferrin saturation, six SNPs associated with ferritin and eight SNPs associated with transferrin at the genome-wide significance threshold (*p* < 5 × 10^−8^) [13] (Table 1). Among those SNPs, three SNPs (rs1800562 and rs1799945 in *HFE* and rs855791 in *TMPRSS6*) showed a robust and consistent association with a systemic iron status and explained the majority of variance for each iron status biomarker [14] and have been used as instrumental variables for iron status in previous MR studies [14,15,16]. The SNPs were uncorrelated (R^2^ < 0.01) and explained 3.4%, 7.2%, 6.9% and 0.9% of the variance for serum iron, transferrin, transferrin saturation and ferritin levels, respectively [13]. Details of the SNPs associated with the iron status biomarkers are presented in Table 1. 

### 2.3. Outcome Data Sources

Summary-level data for breast cancer (including estrogen receptor positive and negative breast cancer) were obtained from the Breast Cancer Association Consortium with 228,951 individuals of European ancestry (122,977 cancer cases and 105,974 controls) [17]. The GWAS for breast cancer used 1000 Genomes Project (Phase 3) reference panel in imputation stage and the logistic regression analyses adjusted for genetic principal components and country. 

Summary-level data for overall cancer and 22 site-specific cancers were extracted from the UK Biobank [18]. This cohort study recruited around 500,000 adults, aged 40 to 69 years, across the UK from 2006 to 2010. To reduce population stratification bias, we confided the study population to European-descent individuals. After exclusion of related individuals (third-degree relatives or closer), low call rate, and excess heterozygosity (3 or more standard deviations from the mean), 367,643 participants remained in the analyses and were followed up until March 31, 2017, or death. In total, there were 75,037 cancer cases at any site.

### 2.4. Statistical Analysis

The inverse-variance weighted method with random-effects was used to assess the causal associations between iron status and cancer risk. In the main analysis, we employed three genetic variants (rs1800562 and rs1799945 in HFE and rs855791 in TMPRSS6), explaining the major proportion of variance for iron status, as instrumental variables. All SNPs reaching the genome-wide significance level for individual iron status indicators were used in the sensitivity analysis. Furthermore, we performed sensitivity analyses, including the weighted median [19] and MR-Egger methods [20], for associations with *p* < 0.05 in the inverse-variance weighted models. Odds ratios (ORs) of cancer are per one standard deviation (SD) increase in genetically predicted serum iron, log10 ferritin, ferritin saturation and transferrin levels in all analyses. Power calculations were based on a method designed for a binary outcome [21]; results from these analyses are displayed in Supplementary Figure S1. All statistical analyses were two-sided and performed in Stata/SE 15.0 and R 3.6.0 software. We searched the PhenoScanner V2 database [22] (a database of human genotype-phenotype associations) to detect possible pleiotropy for the SNPs associated with the iron status biomarkers. The *p* values were not strictly used to define statistical significance, but we interpreted the results based on the strengths of the associations [23] as well as the consistency across sensitivity analyses.

## 3. Results

Genetically predicted high iron status was positively associated with liver cancer and inversely associated with brain cancer but was not associated with overall cancer and the other 20 cancers (Table 2). The OR of liver cancer was 2.45 (95% CI, 0.81, 7.45; *p* = 0.11), 2.11 (95% CI, 1.16, 3.83; *p* = 0.02), 10.89 (95% CI, 2.44, 48.59; *p* = 0.002) and 0.30 (95% CI, 0.17, 0.53; *p* = 2 × 10^−5^) for one SD increase in genetically predicted serum iron, log10 ferritin, ferritin saturation and transferrin levels. Results were consistent across sensitivity analyses (Table 3). Substantial heterogeneity among estimates of individual SNPs (I^2^ = 76, *p* = 0.01) and potential pleiotropy (intercept −0.662 (95% CI -1.124, -0.199); *p* = 5.00 × 10^−3^) were detected in the analysis of serum iron levels. The associations of iron status biomarkers with liver cancer were mainly driven by rs1800562 in *HFE* (Table 3), which had the largest effect on iron status (Table 1). Even though none of the associations remained significant, the magnitude and direction of associations were similar after excluding that SNP (Supplementary Table S2). The OR of brain cancer was 0.69 (95% CI, 0.48, 1.00; *p* = 0.05), 0.75 (95% CI, 0.59, 0.97; *p* = 0.03), 0.41 (95% CI, 0.20, 0.88; *p* = 0.02) and 1.49 (95% CI, 1.04, 2.14; *p* = 0.03) per one SD increment of serum iron, log10 ferritin, ferritin saturation and transferrin levels. The results for brain cancer were consistent and robust in all sensitivity analyses, and no heterogeneity and pleiotropy was observed (Table 4). However, as for liver cancer, the associations did not remain after removing rs1800562 (Supplementary Table S2). 

In the sensitivity analyses using all SNPs for each iron status biomarker, the direction and magnitude of the effects of iron status on liver and brain cancer remained, but only the associations of serum iron levels with brain cancer and of transferrin saturation with liver and brain cancer reached the conventional level of significance (Supplementary Figures S2–S5). The associations did not remain after removing rs1800562 (Supplementary Table S2).

The three SNPs used as instrumental variables for the main analysis were associated with blood cells, HbA1c and blood pressure at genome-wide significance. Rs1800562 in *HFE* was additionally related to some other traits, such as height, lipids and pulse rate (Supplementary Table S3). The other SNPs included in the secondary analysis had pleiotropic associations with multiple traits, such as blood cells, cardiovascular diseases, blood lipids, plasma fatty acids, interleukins, pulse rate, asthma and heart rate.

## 4. Discussion

The present two-sample MR study is the first to systematically evaluate the causal role of iron status for a wide range of cancers. It showed that genetically high iron status was associated with higher risk of liver cancer and lower risk of brain cancer. These associations were driven by rs1800562, which had the largest impact on iron status among the instrumental variables. There was no evidence in support of a causal association of high iron status with overall cancer and 20 other cancers. 

Our results are in line with abundant data from observational and experimental studies, indicating carcinogenic effects of high iron levels on liver cancer. In a recent meta-analysis of cohort and case-control studies, serum ferritin (6 studies) and serum iron (3 studies) levels in the highest category were associated with 1.5- and 2.5-fold increases in risk of liver cancer, respectively, when compared to levels in the lowest group [9]. Moreover, high transferrin saturation (≥60% versus <50%) was reported to be associated with a significant 5.9-fold higher risk of liver cancer in a prospective cohort of 8763 Danish adults followed up for 15 years [24]. In addition, hepatocellular carcinoma risk is substantially increased among individuals with hemochromatosis gene mutations and in hemochromatosis patients diagnosed by elevated serum transferrin saturation and ferritin levels [1,25,26]. Notably, the two HFE SNPs included in this study are known to cause forms of haemochromatosis, with rs1800562 (*C282Y*) causing a particularly serious type, and rs1799945 (*H63D*) causing a milder form [27,28]. These SNPs are related to decreased levels of hepcidin, thereby causing the increase of iron absorption and parenchymal deposition (iron overload) in the liver [29]. Our findings are therefore consistent with previous reports that heterozygosity and combined heterozygosity of these two SNPs are associated with liver cancer. A recent review indicated that iron overload was a common consequence of an excessive and prolonged consumption of alcohol. An increased level of iron was observed in both serum and within cells, hepatocytes in particular [30]. Furthermore, a dose-response relationship has been observed between alcohol consumption and liver cancer [31]. In the present study, SNPs used for iron status were not related to alcohol consumption, implying that the observed association between iron status and liver cancer is unlikely driven by alcohol consumption. Thus, it is speculated that elevated iron status may partly mediate the pathway from alcohol consumption to liver cancer, which is in line with our findings indirectly.

These epidemiologic associations between iron status and liver cancer have been further supported by animal studies. Long-Evans Cinnamon rats fed an iron-deficient diet for 65 weeks exhibit lower risk of fulminant hepatitis, hepatic fibrosis, and subsequent hepatocarcinogenesis compared with those fed a normal diet after a 65-week intervention [32]. Similarly, iron deposition in the liver is likely to promote liver cancer in humans as patients with hepatocellular carcinoma complicating noncirrhotic liver [33] and non-alcoholic steato-hepatitis [34] have higher iron deposition in non-tumorous areas of the liver than those without cancer. A range of putative carcinomic mechanisms of high iron status in the liver have been proposed, including deoxyribonucleic acid damage and cytotoxic by-products of lipid peroxidation caused by reactive oxygen species, free radicals, ferroptosis [26,35,36], facilitation of cancer growth [37], and lymphocyte and macrophage functions impairment [35].

This MR study, therefore, adds strong causal evidence to previous observational and experimental studies that high physiological levels of iron status promote liver cancer. This has important clinical implications with regards to the treatment of borderline anaemia and the continuation of iron therapies in anaemic patients after their iron status has been corrected, particularly if patients are asymptomatic. Conversely, there may be a benefit to careful reduction of iron indices for reducing liver cancer risk in patients with high physiological iron status or in patients who are at risk due to other factors such as alcohol-related liver disease or viral hepatitis. Further studies should address the risk of liver cancer in patients receiving long-term iron therapy with borderline anaemia or normalized iron status. Similarly, the benefits of regular phlebotomy or iron chelation seen with hemochromatosis may potentially be extended in specific groups of patients with high physiological iron levels.

Data on the association between iron status and brain cancer are limited. Nevertheless, iron-related gene expression has been reported to be dysregulated in brain tumours, such as elevated HFE expression in meningioma [38] an iron redox state affects brain cancer severity. These studies suggest that iron homeostasis may be involved in the development of brain cancer, but more broadly, several studies have shown that iron has important effects on the brain. Iron deficiency causes neuropsychologic impairment [39] and behavioural dysfunction [40], and even contributed to long-term organic change in brain, such as dopamine metabolism, myelination, and hippocampal structure and function [40]. Additionally, it has been postulated that both iron excess and deficiency lead to oxidative deoxyribonucleic acid damage, thereby increasing the risk of cancer [3]. Consistent with a protective effect of low iron levels on brain cancer risk, iron chelation with deferoxamine has been suggested as a novel approach to therapy for brain cancer, due to antiproliferative effects, which are mediated by an intracellular pool of iron [37]. Genetic evidence also lends support to our findings stating that the TMPRSS6 gene exerts an important role in the absorption of dietary iron via intestines and transporting out iron from storage sites (particularly in the liver and spleen) to other organs via the bloodstream [41]. Iron status in the brain can be different from other organs due to the presence of the blood-brain barrier. It has been well acknowledged that iron is taken up by means of receptor-mediated uptake of iron-transferrin at the blood-brain barrier, thereby transporting iron from the circulation into the brain extracellular space [42]. Our study showed that both lower serum iron and higher transferrin levels were associated with higher risk of brain cancer. An animal study indicated that iron supplementation prevented lead-induced disruption of the blood–brain barrier in the rat development [43]. Population-based studies have also found that iron deficiency caused alternations of brain development and functioning, possibly also affecting the brain barrier [44]. Blood-brain barrier breakdown or alterations in transport systems have been shown to play a vital role in the pathogenesis of many central nervous system diseases, including tumours [45]. Considering the important role of the blood–brain barrier for iron homeostasis and tumours in the brain along with limited and inconsistent findings of the effects of physiologically high iron levels on blood-brain barrier, additional research on the effects of iron status on blood-brain barrier function and on brain cancer risk is warranted. Although our MR study provides causal evidence of an inverse relationship between iron status and overall brain cancer, considering the small number of brain cancer cases, additional studies with larger sample sizes are warranted to verify our findings. Furthermore, the effects of iron on brain cancer types need to be explored.

A consistent, albeit nonsignificant pattern of high iron status in a high risk of thyroid cancer was observed in the present study. In a study with 102 metastatic thyroid cancer patients, serum ferritin levels were higher in the thyroid cancer group than among primary hypothyroid patients [46]. In addition, a recent cell study found a new pathway of E4BP4/G9a/SOSTDC1/hepcidin linking cellular iron dysfunction to thyroid cancer [47]. The potential role of iron status in the development of thyroid cancer needs further investigation.

Findings of observational studies of the association of iron status with other cancers are conflicting or limited [8,10], but particular attention has related to dietary iron intake and colorectal cancer. A systematic review and meta-analysis found that heme iron intake was positively associated with risk of colorectal and colon cancer, whereas serum iron levels were inversely associated with these cancers [8]. However, the inverse association with serum iron levels may be related to reverse causality as iron deficiency is a common presentation of colon cancer patients [48]. A prospective cohort study of 35,121 US men found no association between frequent blood donations, which may reduce body iron stores, and colorectal cancer incidence or mortality [49]. Our results are, therefore, in agreement with previous observational evidence, which do not support an important role of iron stores in colorectal carcinogenesis. The previously observed findings for heme iron, which is found in meat, poultry, and seafood, may be related to a local adverse effect of heme iron in the colorectum, such as a promoting effect on intestinal N-nitroso compound formation [50]. Another recent meta-analysis found that heme iron intake (based on six studies) and serum iron levels (based on four studies), but no other iron status biomarkers, were significantly positively associated with risk of breast cancer [10]. The present study showed no evidence supporting a causal association between iron status and breast cancer risk. The discrepancy in findings from this MR study and previous observational studies may be attributed to residual confounding from other compounds in meat, unhealthy dietary patterns or other lifestyle behaviours. No consistent association has been reported between iron status and other cancers, but epidemiological data are scarce [8].

A major strength of this study is the MR study design, which minimizes the confounding and reverse causality seen in observational studies. In addition, we systematically assessed the associations between four individual iron status indicators and 22 cancers using summary-level data from large-scale genetic consortia and cohorts. The studies only used data from European populations. This reduced population stratification bias but confined the transferability of our findings to other populations. A major limitation of this study is that the statistical power was low in several analyses due to a small number of cases for most cancer sites. In addition, this MR study investigated the association of iron status within the normal range with cancer risk. Therefore, our results cannot be used to make inferences regarding the effect of abnormally high serum iron levels caused by, for example, intravenous, long-term oral iron supplementation or hemochromatosis.

## 5. Conclusions

The present study showed that genetically predicted iron status was positively associated with risk of liver cancer and inversely associated with risk of brain cancer but not associated with overall cancer and a number of other site-specific cancers among European-descent individuals. These findings need to be interpreted with caution in light of the influential effect of a single variant in the HFE gene and the limited number of liver and brain cancer cases. Nevertheless, our results, along with observational data, highlight the importance of further research on the impact of adjustment of iron levels within the physiological range, through supplementation or chelation, on risk of these cancers types. In particular, careful consideration may be needed with regards to ongoing supplementation in anaemic patients or those at high risk of liver cancer.

## Figures and Tables

**Table 1:** SNPs associated with iron status biomarkers at genome-wide significance level and included in the main analyses with three primary SNPs and secondary analyses of all SNPs.

SNP	Nearby Gene	EA	EAF	Serum Iron, μmol/L			Transferrin Saturation, %			Log10 Ferritin, μg/L			Ferritin, g/L		
				Beta	SE	*p*	Beta	SE	*p*	Beta	SE	*p*	Beta	SE	*p*
rs1800562 *	*HFE*	A	0.07	0.328	0.016	2.9 × 10^−97^	0.577	0.016	2.2 × 10^−270^	0.204	0.016	1.5 × 10^−38^	-0.479	0.016	8.9 × 10^−196^
rs1799945 *	*HFE*	G	0.15	0.189	0.010	1.1 × 10^−81^	0.231	0.010	5.1 × 10^−109^	0.065	0.010	1.7 × 10^−10^	-0.114	0.010	9.4 × 10^−30^
rs855791 *	*TMPRSS6*	G	0.55	0.181	0.007	4.3 × 10^−139^	0.190	0.007	6.4 × 10^−137^	0.055	0.007	1.4 × 10^−14^	-0.044	0.007	2.0 × 10^−9^
rs8177240	*TF*	G	0.35	0.066	0.007	6.6 × 10^−20^	0.100	0.008	7.2 × 10^−38^				0.380	0.007	8.4 × 10^−610^
rs7385804	*TFR2*	A	0.62	0.064	0.007	1.4 × 10^−18^	0.054	0.008	6.1 × 10^−12^						
rs744653	*AC013439.4*	C	0.16							0.089	0.010	8.4 × 10^−19^			
rs411988	*TEX14*	G	0.44							0.044	0.007	1.6 × 10^−10^			
rs651007	*ABO*	C	0.79							0.050	0.009	1.3 × 10^−8^			
rs4921915	*NAT2*	A	0.76										0.079	0.009	7.1 × 10^−19^
rs174577	*FADS2*	A	0.36										0.062	0.007	2.3 × 10^−17^
rs9990333	*TFRC*	C	0.53										0.051	0.007	2.0 × 10^−13^
rs6486121	*ARNTL*	C	0.34										0.046	0.007	3.9 × 10^−10^

EA indicates effect allele; EAF, effect allele frequency; SE, standard error; SNP, single nucleotide polymorphism. * Three SNPs were included in the main analyses.

**Table 2:** Associations between genetically predicted iron status, based on three primary SNPs, and overall cancer and 22 site-specific cancers.

Cancer	Cases	Serum Iron		Transferrin Saturation		Ferritin		Transferrin	
		OR (95% CI)	*p*	OR (95% CI)	*p*	OR (95% CI)	*p*	OR (95% CI)	*p*
Overall cancer (UKBB)	75,037	1.03 (0.96, 1.10)	0.47	1.01 (0.96, 1.07)	0.65	1.03 (0.88, 1.21)	0.71	1.00 (0.92, 1.08)	0.99
Brain and head and neck									
Brain	810	0.69 (0.48, 1.00)	0.05	0.75 (0.59, 0.97)	0.03	0.41 (0.20, 0.88)	0.02	1.49 (1.04, 2.14)	0.03
Head and neck	1615	0.93 (0.71, 1.23)	0.63	0.97 (0.79, 1.19)	0.77	0.93 (0.50, 1.72)	0.81	0.99 (0.73, 1.34)	0.95
Gastrointestinal tract									
Oesophagus	843	0.95 (0.68, 1.33)	0.76	0.95 (0.75, 1.20)	0.66	0.82 (0.40, 1.71)	0.60	1.11 (0.78, 1.57)	0.56
Stomach	736	1.11 (0.67, 1.83)	0.70	1.00 (0.75, 1.35)	0.97	1.00 (0.41, 2.46)	0.99	1.06 (0.69, 1.61)	0.80
Colorectum	5486	0.99 (0.70, 1.41)	0.95	0.98 (0.76, 1.26)	0.89	0.95 (0.44, 2.04)	0.90	1.06 (0.74, 1.51)	0.76
Pancreas	1264	1.03 (0.72, 1.48)	0.86	1.04 (0.81, 1.34)	0.76	1.17 (0.55, 2.52)	0.68	0.92 (0.64, 1.32)	0.65
Liver	324	2.45 (0.81, 7.45)	0.11	2.11 (1.16, 3.83)	0.01	10.89 (2.44, 48.59)	2.0×10^−3^	0.30 (0.17, 0.53)	2.0×10^−5^
Biliary tract	387	0.67 (0.34, 1.32)	0.25	1.06 (0.65, 1.72)	0.81	1.33 (0.31, 5.63)	0.70	0.77 (0.42, 1.43)	0.41
Sex-specific									
Breast (UKBB)	13,666	1.10 (1.00, 1.22)	0.05	1.06 (0.98, 1.16)	0.16	1.18 (0.89, 1.56)	0.25	0.94 (0.81, 1.10)	0.47
Breast (BCAC)	122,977	0.99 (0.94, 1.03)	0.62	0.99 (0.96, 1.03)	0.65	0.98 (0.88, 1.08)	0.66	1.01 (0.96, 1.06)	0.75
Breast ER+ (BCAC)	69,501	1.01 (0.96, 1.07)	0.73	1.00 (0.97, 1.03)	0.85	1.03 (0.91, 1.17)	0.63	0.98 (0.93, 1.05)	0.60
Breast ER- (BCAC)	21,468	0.93 (0.85, 1.01)	0.07	0.95 (0.89, 1.01)	0.08	0.85 (0.70, 1.02)	0.09	1.07 (0.98, 1.18)	0.14
Uterus	1931	0.99 (0.79, 1.24)	0.95	0.98 (0.84, 1.15)	0.83	0.94 (0.58, 1.53)	0.80	1.06 (0.83, 1.33)	0.65
Cervix	1928	1.11 (0.88, 1.41)	0.38	1.06 (0.88, 1.27)	0.53	1.18 (0.67, 2.08)	0.57	0.97 (0.73, 1.30)	0.84
Ovary	1520	0.98 (0.69, 1.39)	0.91	0.97 (0.76, 1.24)	0.82	0.92 (0.43, 1.94)	0.82	1.08 (0.76, 1.53)	0.66
Prostate	7872	1.10 (0.91, 1.33)	0.34	1.07 (0.94, 1.22)	0.32	1.24 (0.83, 1.85)	0.29	0.91 (0.74, 1.12)	0.40
Testis	735	1.11 (0.77, 1.60)	0.57	1.11 (0.86, 1.44)	0.43	1.42 (0.64, 3.12)	0.39	0.81 (0.56, 1.19)	0.29
Urinary tract									
Bladder	2588	1.08 (0.89, 1.31)	0.46	1.045 (0.91, 1.2)	0.51	1.15 (0.75, 1.76)	0.52	0.96 (0.78, 1.17)	0.67
Kidney	1310	0.97 (0.73, 1.28)	0.82	1.01 (0.82, 1.23)	0.95	1.07 (0.58, 1.96)	0.83	0.93 (0.70, 1.23)	0.60
Blood/bone marrow/lymph									
Leukemia	1403	0.99 (0.77, 1.29)	0.96	0.98 (0.81, 1.18)	0.82	0.91 (0.51, 1.61)	0.75	1.07 (0.81, 1.40)	0.65
Non-Hodgkin lymphoma	2296	0.92 (0.75, 1.13)	0.43	0.95 (0.82, 1.10)	0.50	0.87 (0.55, 1.36)	0.53	1.05 (0.84, 1.30)	0.67
Multiple myeloma	656	0.80 (0.41, 1.59)	0.53	0.80 (0.60, 1.08)	0.15	0.51 (0.21, 1.22)	0.13	1.33 (0.82, 2.14)	0.25
Other									
Thyroid	375	1.68 (0.63, 4.49)	0.30	1.54 (0.81, 2.93)	0.19	4.06 (0.65, 25.53)	0.14	0.51 (0.21, 1.24)	0.14
Lung	2838	0.93 (0.77, 1.12)	0.43	0.93 (0.82, 1.07)	0.31	0.80 (0.54, 1.20)	0.29	1.13 (0.94, 1.37)	0.20
Melanoma	4869	0.96 (0.83, 1.11)	0.58	0.98 (0.88, 1.08)	0.66	0.94 (0.69, 1.28)	0.71	1.01 (0.87, 1.18)	0.86

BCAC indicates Breast Cancer Association Consortium; CI; confidence interval; ER, estrogen receptor; OR, odds ratio; UKBB, UK Biobank. All estimations were based on the inverse-variance weighted method with random-effects.

**Table 3:** Associations between genetically predicted iron status, based on three primary SNPs, and liver cancer in sensitivity analyses.

SNP (Gene) or Method	Serum Iron		Transferrin Saturation		Ferritin		Transferrin	
	OR (95% CI)	*p*	OR (95% CI)	*p*	OR (95% CI)	*p*	OR (95% CI)	*p*
rs1800562 (*HFE*)	6.65 (2.78, 15.9)	2.02 × 10^−5^	2.94 (1.79, 4.82)	2.02 × 10^−5^	21.0 (5.18, 85.4)	2.02 × 10^−5^	0.27 (0.15, 0.50)	2.02 × 10^−5^
rs1799945 (*HFE*)	1.01 (0.32, 3.16)	0.99	1.01 (0.40, 2.56)	0.99	1.03 (0.04, 28.3)	0.99	0.98 (0.15, 6.50)	0.99
rs855791 (*TMPRSS6)*	1.54 (0.65, 3.64)	0.32	1.51 (0.67, 3.42)	0.32	4.14 (0.24, 70.3)	0.32	0.17 (0.01, 5.82)	0.32
IVW-Random effects	2.45 (0.81, 7.45)	0.11	2.11 (1.16, 3.83)	0.01	10.9 (2.44, 48.6)	2.00 × 10^−3^	0.30 (0.17, 0.53)	2.99 × 10^−5^
Weighted median	2.08 (0.97, 4.47)	0.06	2.14 (1.39, 3.31)	1.00 × 10^−3^	12.1 (3.24, 45.2)	3.43 × 10^−4^	0.30 (0.17, 0.53)	3.31 × 10^−5^
MR-Egger	49.0 (5.64, 424)	4.17 × 10^−4^	4.36 (1.87, 10.1)	1.00 × 10^−3^	46.4 (5.47, 394)	4.38 × 10^−4^	0.28 (0.11, 0.70)	7.00 × 10^−3^
Heterogeneity (I^2^)	76 (23, 93)	0.01	58 (0, 88)	0.09	38 (0, 81)	0.20	0 (0, 90)	0.43
Pleiotropy (Intercept)	NA	5.00 × 10^−3^	NA	0.06	NA	0.11	NA	0.76

CI indicates confidence interval; IVW, inverse-variance weighted; NA, Not Available; OR, odds ratio; SNP, single nucleotide polymorphism.

**Table 4:** Associations between genetically predicted iron status, based on 3 primary SNPs, and brain cancer in sensitivity analyses.

SNP (Gene) or Method	Serum Iron		Transferrin Saturation		Ferritin		Transferrin	
	OR (95% CI)	*p*	OR (95% CI)	*p*	OR (95% CI)	*p*	OR (95% CI)	*p*
rs1800562 (*HFE*)	1.09 (0.53, 2.24)	0.82	1.07 (0.59, 1.93)	0.82	1.28 (0.16, 10.4)	0.82	5.04 (0.54, 47.4)	0.82
rs1799945 (*HFE*)	0.54 (0.31, 0.95)	0.16	0.69 (0.41, 1.16)	0.16	0.27 (0.05, 1.65)	0.16	0.87 (0.26, 2.88)	0.16
rs855791 (*TMPRSS6)*	0.67 (0.39, 1.16)	0.03	0.71 (0.51, 0.97)	0.03	0.37 (0.15, 0.92)	0.03	1.52 (1.04, 2.23)	0.03
IVW-Random effects	0.69 (0.48, 1.00)	0.05	0.75 (0.59, 0.97)	0.03	0.41 (0.20, 0.88)	0.02	1.49 (1.04, 2.14)	0.03
Weighted median	0.65 (0.44, 0.96)	0.03	0.71 (0.53, 0.93)	0.02	0.37 (0.16, 0.83)	0.02	1.46 (1.01, 2.13)	0.05
MR-Egger	0.35 (0.07, 1.63)	0.18	0.68 (0.36, 1.29)	0.24	0.36 (0.08, 1.69)	0.19	1.38 (0.74, 2.57)	0.31
Heterogeneity (I^2^)	11 (0, 91)	0.33	0 (0, 90)	0.44	0 (0, 90)	0.51	0 (0, 90)	0.38
Pleiotropy (Intercept)	NA	0.37	NA	0.72	NA	0.82	NA	0.72

CI indicates confidence interval; IVW, inverse-variance weighted; NA, Not Available; OR, odds ratio; SNP, single nucleotide polymorphism.

## Supplementary Materials

The following are available online at https://www.mdpi.com/2072-6643/12/2/526/s1, Figure S1: Power estimation based on phenotypic variance explained and case number, Figure S2: Association between genetically predicted serum iron levels and cancer using all SNPs (*n* = 5), Figure S3: Association between genetically predicted transferrin saturation and cancer using all SNPs (*n* = 5), Figure S4, Association between genetically predicted log10 ferritin and cancer using all SNPs (*n* = 6), Figure S5, Association between genetically predicted transferrin and cancer using all SNPs (*n* = 8), Table S1: Information of included studies and consortia, Table S2, Associations of iron status with liver and brain cancer excluding rs1800562, Table S3, Associations of the instrumental variables for iron status with other traits at the genome-wide significance level.
