# Supplementary material for: Iron Status and Cancer Risk in UK Biobank: A Two-Sample Mendelian Randomization Study
Source: Nutrients. 2020 Feb 19;12(2):526. doi: 10.3390/nu12020526 (PMC7071358; doi:10.3390/nu12020526)
Supplement: Supplementary file 1 [file nutrients-12-00526-s001.pdf]

Supplements

Supplementary table 1. Information of included studies and consortia

Exposure/Outcome	Consortium or cohort study	Participants	Web source if publicly available
Iron status	Genetics of Iron Status Consortium	48 972 European-descent individuals	Not available
Breast cancer	The Breast Cancer Association Consortium	228 951 European-descent individuals, including 122 977 all breast cancer cases (69 501 ER+ and 21 468 ER-) and 105 974 controls	http://bcac.ccge.medschl.cam.ac.uk/
22 cancers	UK Biobank	367 643 unrelated European-descent individuals	https://www.ukbiobank.ac.uk/

Supplementary table 2. Associations of iron status with liver and brain cancer excluding rs1800562

Iron status	OR (95 CI) [a]	*P*	OR (95 CI) [b]	*P*
Liver cancer				
Serum iron	1.20 (0.63, 2.27)	0.578	1.32 (0.67, 2.63)	0.426
Ferritin saturation	1.18 (0.67, 2.08)	0.557	1.27 (0.68, 2.34)	0.453
Ferritin	0.86 (0.22, 3.42)	0.828	2.30 (0.27, 19.8)	0.447
Transferrin	0.88 (0.59, 1.32)	0.545	0.67 (0.13, 3.52)	0.426
Brain cancer				
Serum iron	0.78 (0.51, 1.17)	0.234	0.80 (0.52, 1.24)	0.321
Ferritin saturation	0.84 (0.59, 1.21)	0.350	0.83 (0.56, 1.23)	0.362
Ferritin	1.24 (0.52, 2.97)	0.632	0.52 (0.13, 2.05)	0.354
Transferrin	1.10 (0.86, 1.41)	0.459	1.28 (0.45, 3.69)	0.642

CI indicates confidence interval; OR, odds ratio.

[a] Estimation was based on all SNPs for each iron trait using inverse-variance weighted method with fixed-effects.

[b] Estimation was based on two SNPs (rs1799945 in *HFE* and rs855791 in *TMPRSS6*) using inverse-variance weighted method with fixed-effects.

Supplementary table 3. Associations of the instrumental variables for iron status with other traits at the genome-wide significance level

SNP	Chr	Gene	EA	Trait	Beta	*p* value	Trait	Beta	*p* value
rs1800562	6	*HFE*	A	Mean corpuscular hemoglobin	0.300	0	HbA1c	-0.040	4.7×10^{-28}
				Red cell distribution width	-0.192	7.7×10^{-200}	Diastolic blood pressure	0.394	9.0×10^{-17}
				Reticulocyte count	0.109	1.1×10^{-63}	Low density lipoprotein	-0.062	8.3×10^{-14}
				Erythrocyte indices	NA	1.0×10^{-46}	Height	0.022	6.3×10^{-12}
				Pulse rate	0.031	6.8×10^{-11}			
rs1799945	6	*HFE*	G	Mean corpuscular hemoglobin	0.193	0	HbA1c	-0.021	3.7×10^{-19}
				Red cell distribution width	-0.133	2.8×10^{-161}	Blood pressure	NA	2.0×10^{-15}
				Reticulocyte count	0.057	2.0×10^{-30}	Platelet count	-0.035	5.0×10^{-12}
				Hypertension	NA	2.0×10^{-10}			
rs855791	22	*TMPRSS6*	G	Mean corpuscular hemoglobin	0.170	0	HbA1c	-0.017	3.4×10^{-28}
				Red cell distribution width	0.125	2.8×10^{-271}	Platelet count	-0.032	2.2×10^{-18}
				Reticulocyte count	0.042	8.2×10^{-31}			
rs411988	17	*TEX14*	G	Monocyte % of white cells	-0.035	1.7×10^{-22}	Granulocyte % of myeloid white cells	0.032	1.9×10^{-19}
rs651007	9	*ABP*	C	Blood protein levels	-0.944	1.0×10^{-96}	Hemoglobin concentration	0.072	9.0×10^{-61}
				Serum alkaline phosphatase levels	-0.079	1.0×10^{-56}	Red blood cell count	0.067	1.1×10^{-52}
				deep venous thrombosis	-0.006	6.0×10^{-50}	Blood clot in the lung	-0.003	3.7×10^{-34}
				Total cholesterol	NA	1.0×10^{-21}	Low density lipoprotein	-0.066	4.5×10^{-21}
				Monocyte count	0.039	5.7×10^{-19}	Plasma carcinoembryonic antigen levels	NA	2.1×10^{-18}
				Granulocyte count	0.036	4.9×10^{-16}	Myocardial infarction	-0.103	6.7×10^{-16}
				Coronary artery disease	-0.053	3.6×10^{-14}	Hemoglobin Hb	NA	3.8×10^{-14}
				Interleukin 6	NA	3.4×10^{-12}	High grade serous ovarian cancer	-0.113	2.7×10^{-9}
				Arm fat percentage	-0.012	1.3×10^{-8}			
rs4921915	8	*NAT2*	A	Triglycerides	-0.035	1.3×10^{-15}	Total cholesterol	-0.032	6.7×10^{-13}
rs174577	11	*FADS2*	A	Arachidonic acid	-1.685	0	Linoleic acid	1.463	1.7×10^{-263}
				Dihomo-gamma-linolenic acid	0.358	3.4×10^{-152}	Docosapentaenoic acid levels	-0.075	2.7×10^{-149}
				Adrenic acid	-0.048	9.1×10^{-134}	Red cell distribution width	-0.050	6.3×10^{-41}
				Low density lipoprotein	-0.052	1.0×10^{-40}	Total cholesterol	-0.049	1.1×10^{-37}
				Pulse rate	0.033	1.4×10^{-35}	Triglycerides	0.043	7.6×10^{-35}
				High density lipoprotein	-0.039	9.7×10^{-27}	Platelet count	0.037	9.1×10^{-22}

				Red blood cell count	0.034	2.6×10^{-20}	Fasting glucose	-0.020	1.3×10^{-18}
				Height	-0.013	3.1×10^{-12}	Self-reported asthma	-0.005	4.3×10^{-10}
				Neutrophil count	-0.023	8.2×10^{-10}	Eosinophil count	-0.023	8.6×10^{-10}
				White blood cell count	-0.022	3.2×10^{-9}	Reticulocyte count	0.021	1.3×10^{-8}
				Heart rate	0.314	4.9×10^{-8}			
rs9990333	3	*TFRC*	C	Mean corpuscular hemoglobin	0.056	2.6×10^{-55}	Red cell distribution width	-0.046	1.3×10^{-37}
				Red blood cell count	-0.031	1.5×10^{-17}			

Chr indicates chromosome; EA, effect allele; SNP, single nucleotide polymorphism.
The link of PhenoScanner V2: http://www.phenoscanner.medschl.cam.ac.uk/
Related or repeated traits were counted once.

Supplementary figure 1. Power estimation based on phenotypic variance explained and case number*

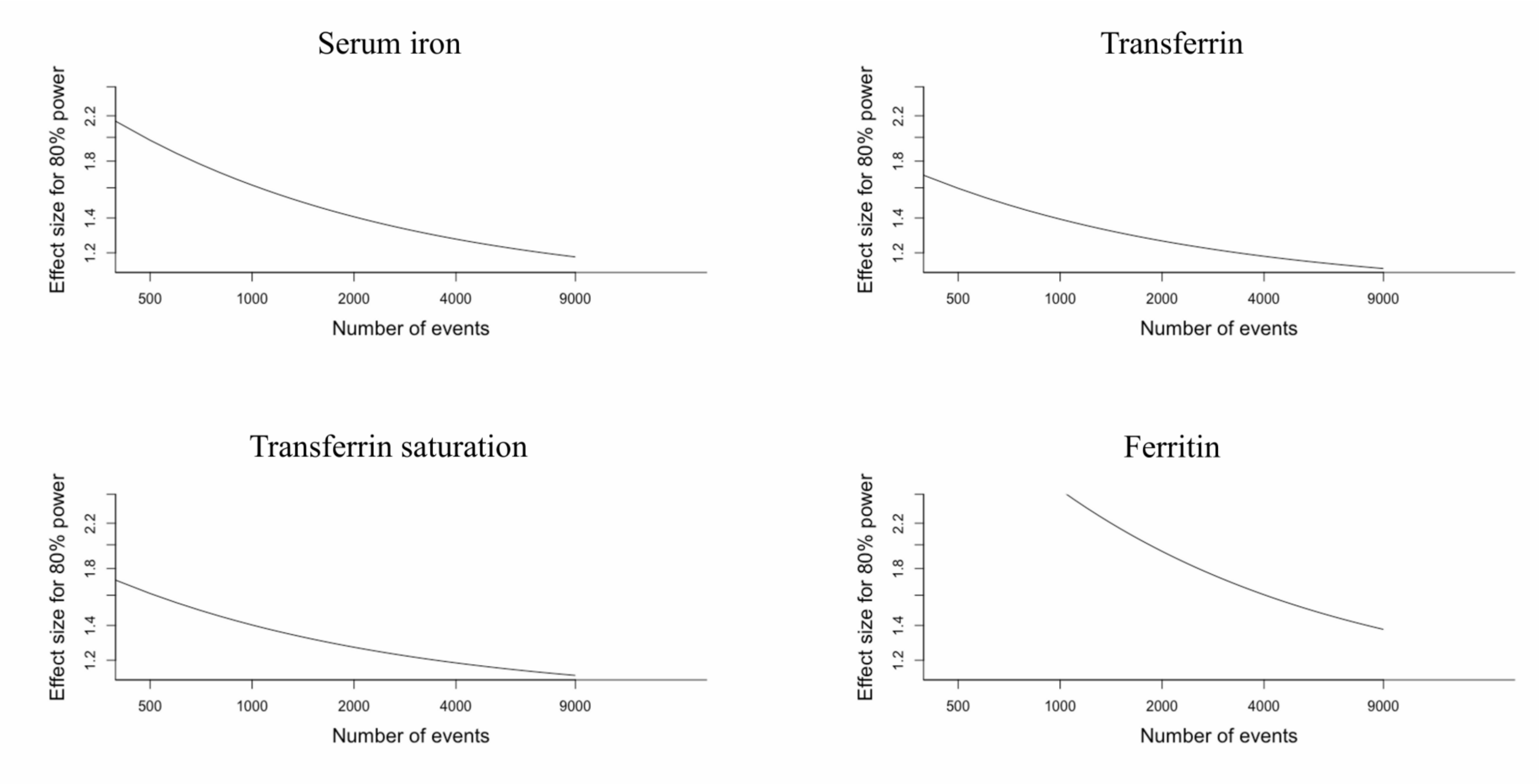

*The significance level was set at 0.05 and the variance explained by the genetic instruments was 3.4%, 7.2%, 6.9% and 0.9% for serum iron, transferrin, transferrin saturation and ferritin, respectively.

Supplementary figure 2. Association between genetically predicted serum iron levels and cancer using all SNPs (n=5)

Cancer site	Cases	OR (95% CI)	P
Brain	810	0.69 (0.50, 0.96)	0.026
Biliary tract	387	0.77 (0.33, 1.82)	0.552
Multiple myeloma	656	0.84 (0.46, 1.55)	0.580
Non-Hodgkin lymphoma	2296	0.90 (0.74, 1.10)	0.323
Lung	2838	0.91 (0.77, 1.09)	0.311
Head-neck	1615	0.93 (0.74, 1.17)	0.545
Ovary	1520	0.93 (0.71, 1.24)	0.635
Breast_erneg	21468	0.94 (0.86, 1.01)	0.105
Oral cavity	960	0.94 (0.69, 1.26)	0.670
Melanoma	4869	0.95 (0.83, 1.08)	0.426
Leukemia	1403	0.96 (0.74, 1.23)	0.724
Kidney	1310	0.99 (0.72, 1.36)	0.944
Breast_all	122977	0.99 (0.92, 1.06)	0.789
Oesophagus	843	0.99 (0.72, 1.37)	0.962
Breast_erpos	69501	1.00 (0.95, 1.07)	0.871
Bowel	5486	1.02 (0.77, 1.35)	0.896
Uterus	1931	1.03 (0.83, 1.27)	0.815
Stomach	736	1.05 (0.69, 1.61)	0.807
Bladder	2588	1.07 (0.89, 1.29)	0.464
Cervix	1928	1.07 (0.87, 1.33)	0.520
Pancreas	1264	1.09 (0.78, 1.53)	0.598
Prostate	7872	1.10 (0.95, 1.27)	0.191
Testis	735	1.18 (0.84, 1.67)	0.340
Larynx	448	1.23 (0.66, 2.27)	0.513
Thyroid	375	1.61 (0.81, 3.21)	0.174
Liver	324	2.18 (0.94, 5.08)	0.070

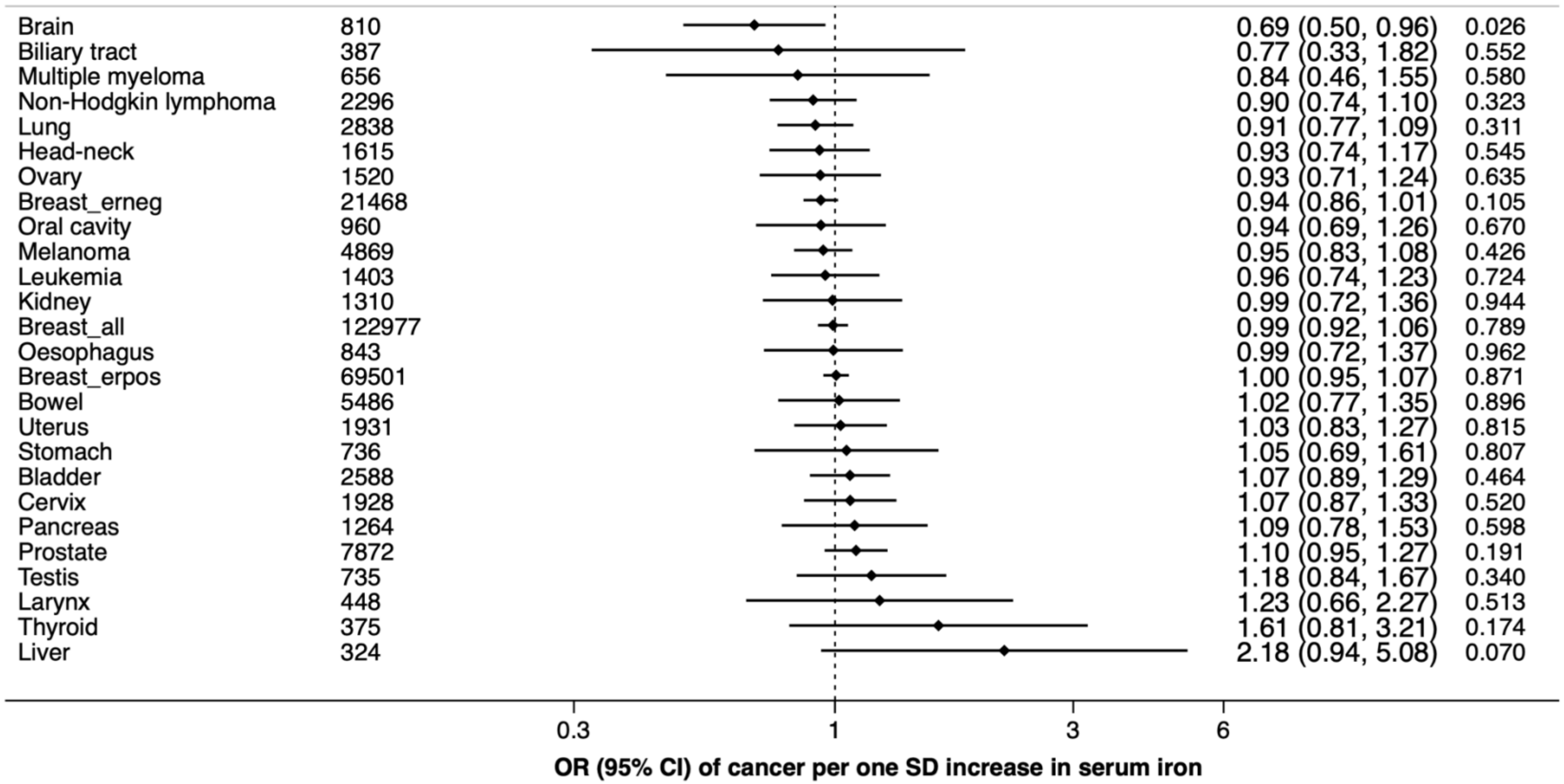

CI indicates confidence interval; OR, odds ratio; SD, standard deviation.

Supplementary figure 3. Association between genetically predicted transferrin saturation and cancer using all SNPs (n=5)

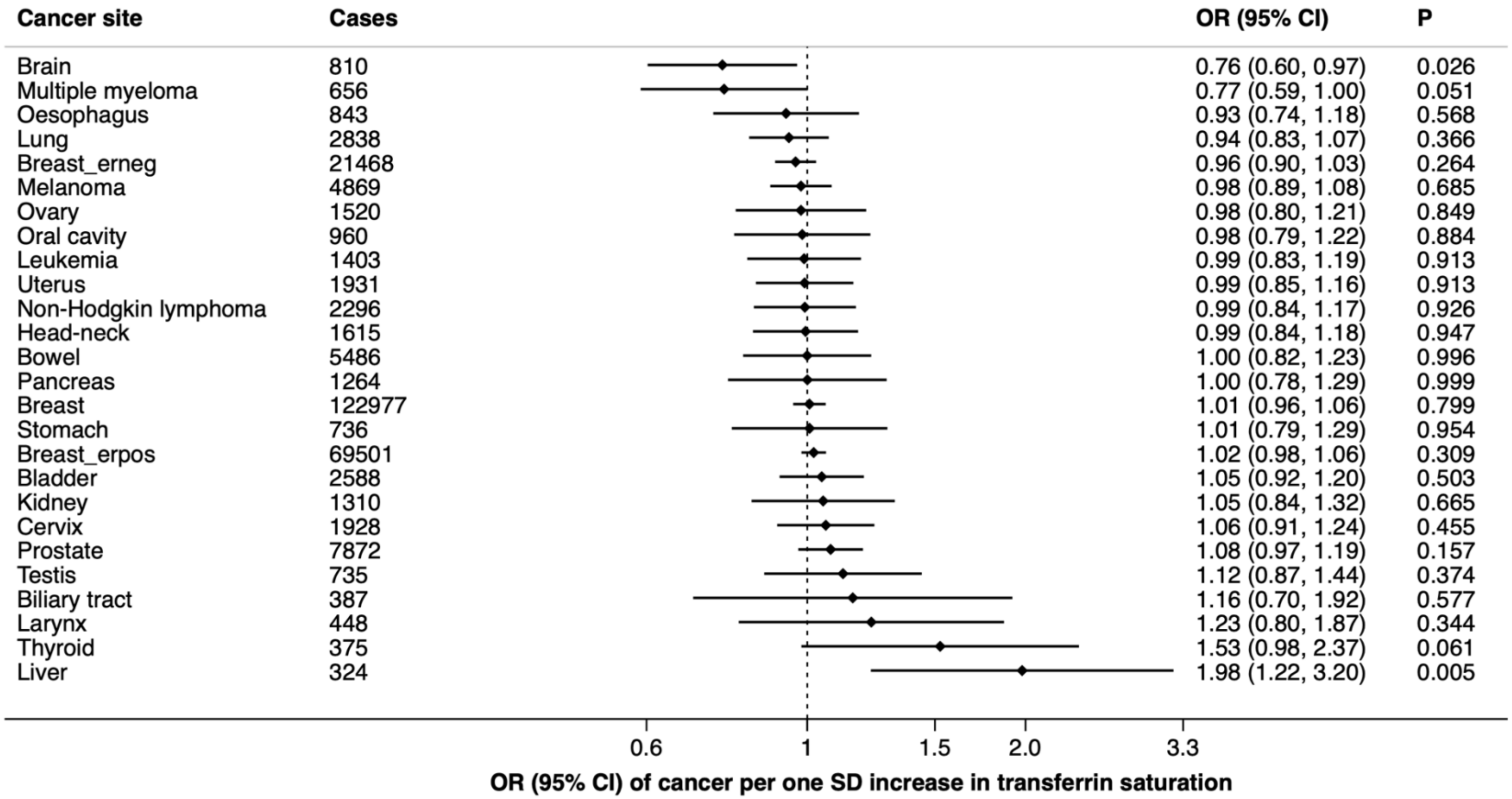

CI indicates confidence interval; OR, odds ratio; SD, standard deviation.

Supplementary figure 4. Association between genetically predicted log_{10} ferritin and cancer using all SNPs (n=6)

Cancer site	Cases	OR (95% CI)	P
Brain	810	0.69 (0.30, 1.61)	0.392
Multiple myeloma	656	0.76 (0.33, 1.74)	0.512
Ovary	1520	0.76 (0.41, 1.44)	0.409
Oesophagus	843	0.86 (0.39, 1.89)	0.712
Bowel	5486	0.86 (0.54, 1.38)	0.539
Leukemia	1403	0.90 (0.56, 1.44)	0.651
Lung	2838	0.90 (0.58, 1.40)	0.646
Oral cavity	960	0.91 (0.49, 1.70)	0.765
Breast_erneg	21468	0.94 (0.73, 1.20)	0.610
Non-Hodgkin lymphoma	2296	0.97 (0.67, 1.42)	0.891
Breast_all	122977	0.98 (0.79, 1.22)	0.856
Uterus	1931	0.99 (0.64, 1.53)	0.972
Breast_erpos	69501	1.00 (0.80, 1.24)	0.985
Stomach	736	1.00 (0.41, 2.42)	0.998
Pancreas	1264	1.02 (0.50, 2.07)	0.952
Melanoma	4869	1.03 (0.80, 1.33)	0.814
Testis	735	1.05 (0.54, 2.03)	0.884
Bladder	2588	1.08 (0.76, 1.54)	0.671
Head-neck	1615	1.08 (0.57, 2.04)	0.811
Biliary tract	387	1.16 (0.47, 2.87)	0.748
Prostate	7872	1.18 (0.86, 1.62)	0.297
Kidney	1310	1.32 (0.75, 2.31)	0.331
Cervix	1928	1.42 (0.94, 2.13)	0.094
Larynx	448	1.97 (0.67, 5.81)	0.221
Liver	324	4.16 (0.90, 19.23)	0.068
Thyroid	375	4.39 (1.52, 12.69)	0.006

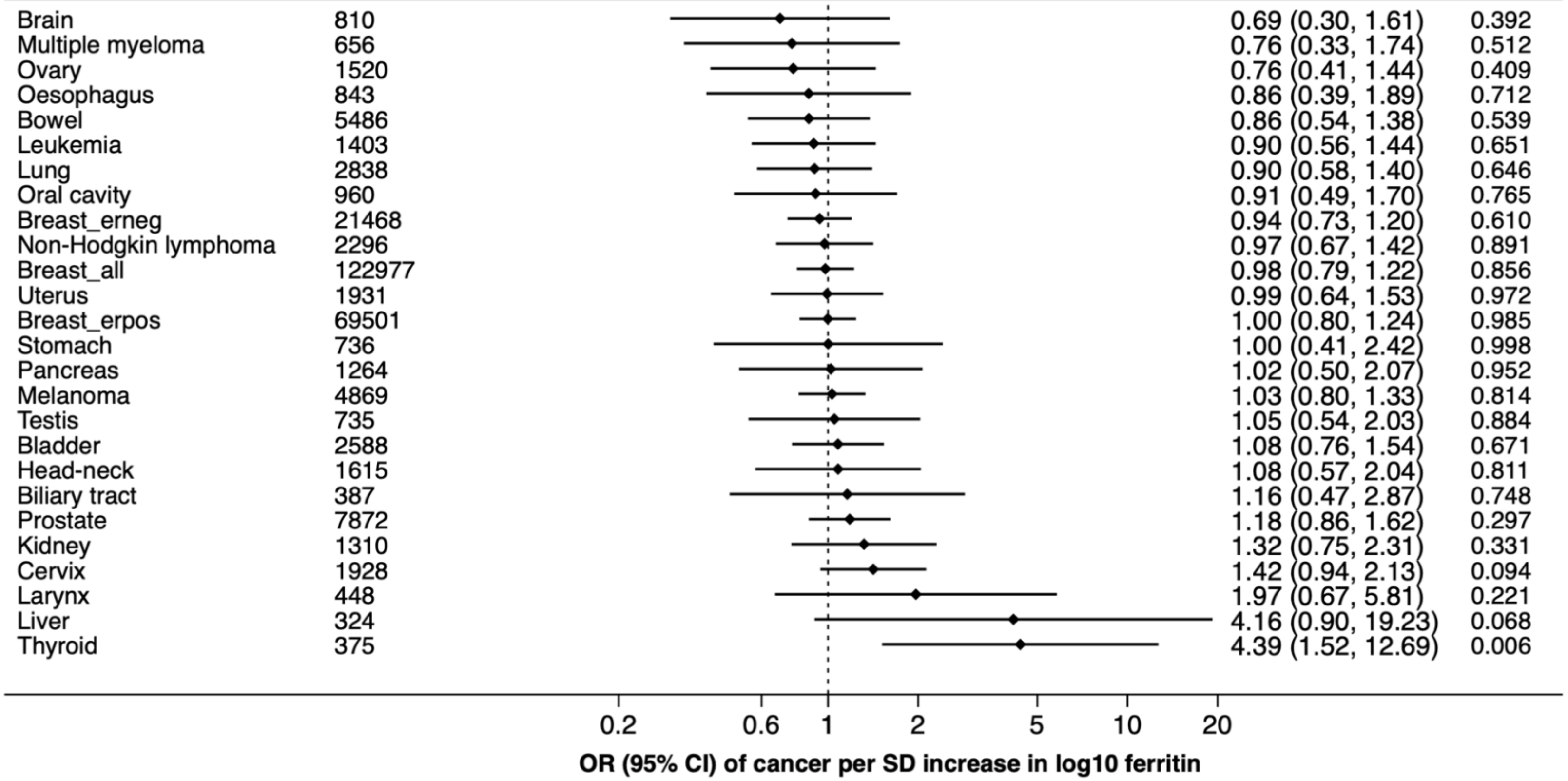

CI indicates confidence interval; OR, odds ratio; SD, standard deviation.

Supplementary figure 5. Association between genetically predicted transferrin and cancer using all SNPs (n=8)

Cancer site	Cases	OR (95% CI)	P
Liver	324	0.62 (0.36, 1.06)	0.081
Thyroid	375	0.76 (0.54, 1.09)	0.136
Biliary tract	387	0.82 (0.61, 1.11)	0.201
Larynx	448	0.82 (0.58, 1.16)	0.262
Kidney	1310	0.90 (0.74, 1.09)	0.288
Non-Hodgkin lymphoma	2296	0.92 (0.81, 1.05)	0.214
Head-neck	1615	0.93 (0.77, 1.14)	0.507
Oral cavity	960	0.94 (0.71, 1.24)	0.651
Prostate	7872	0.95 (0.86, 1.05)	0.297
Ovary	1520	0.96 (0.82, 1.12)	0.577
Cervix	1928	0.96 (0.84, 1.10)	0.565
Stomach	736	0.97 (0.75, 1.25)	0.815
Breast_erpos	69501	0.97 (0.94, 1.00)	0.097
Melanoma	4869	0.98 (0.90, 1.06)	0.597
Breast_all	122977	0.98 (0.95, 1.01)	0.156
Leukemia	1403	0.98 (0.84, 1.15)	0.808
Bowel	5486	0.99 (0.85, 1.14)	0.861
Breast_erneg	21468	0.99 (0.92, 1.07)	0.803
Lung	2838	1.00 (0.85, 1.16)	0.962
Bladder	2588	1.00 (0.83, 1.21)	0.981
Testis	735	1.00 (0.80, 1.25)	0.996
Uterus	1931	1.03 (0.90, 1.18)	0.674
Oesophagus	843	1.05 (0.82, 1.34)	0.692
Pancreas	1264	1.10 (0.87, 1.40)	0.432
Brain	810	1.21 (0.92, 1.59)	0.166
Multiple myeloma	656	1.23 (0.97, 1.55)	0.088

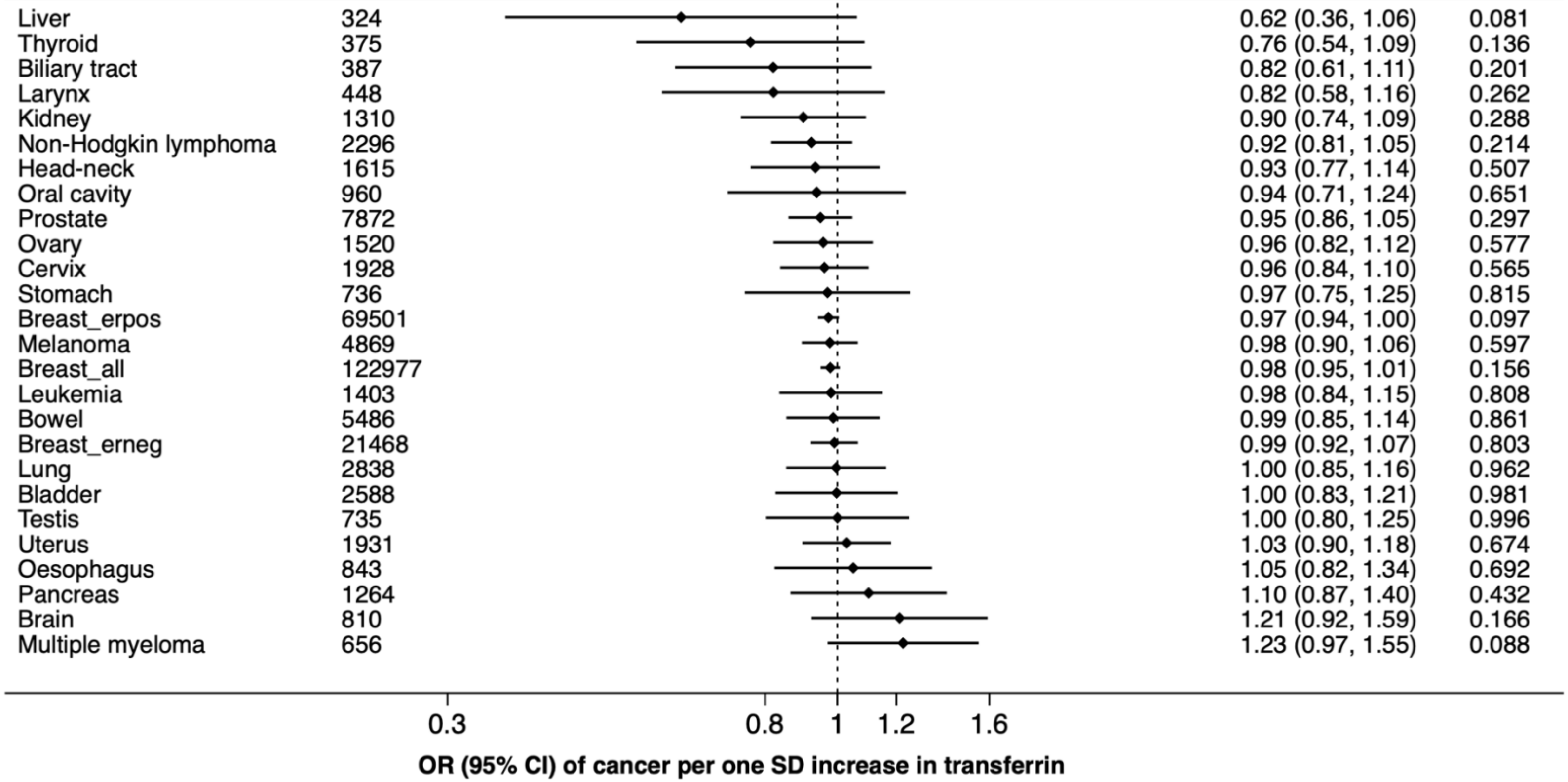

CI indicates confidence interval; OR, odds ratio; SD, standard deviation.
